# Natural Antioxidant in Cardiovascular and Cerebrovascular Diseases

**DOI:** 10.3390/antiox11061159

**Published:** 2022-06-13

**Authors:** Yi-Sook Jung

**Affiliations:** College of Pharmacy, Research Institute of Pharmaceutical Sciences and Technology, Ajou University, Suwon 16499, Korea; yisjung@ajou.ac.kr

Cardiovascular (CVD) and cerebrovascular diseases, with 17.9 million and 2 million deaths every year, respectively, are the leading cause of death worldwide [1,2]. The accumulation of reactive oxygen species (ROS) plays a critical role in the pathogenesis of various diseases, including hypertension, post-myocardial infarction, heart failure, atherosclerosis, arrhythmia, stroke, and traumatic brain injury. The homeostatic balance between ROS production and the antioxidant system is essential to prevent the accumulation of oxidative stress and the harmful effects on vascular function. Woo et al. explained that cardiac myocytes produce several ROS-producing pathways causing cardiac stress and eventually leading to cardiovascular dysfunctions; therefore, natural antioxidants may be beneficial to prevent such oxidative-stress-induced cardiac diseases [3]. Lee et al. gave us an insight into the role of microRNAs in CVD pathology and by targeting the endothelial nitric oxide synthase and sirtuin 1 through the consumption of natural antioxidant [4]. Uddin et al. gave an in-depth analysis of the correlation between chronic kidney disease (CKD) and oxidative stress. They also suggest that nuclear-factor-erythroid-2-related factor 2-heme oxygenase-1 (NRF2-HO-1) signaling might be a possible target for the treatment of CKD, as well as the potential of natural products against CKD [5]. Kang et al. have elucidated the importance of NRF2 in targeting cardiac fibrosis by docosahexanoic acid (DHA) and eicosapentaenoic acid (EPA) and the novel use of specialized pro-resolving lipid mediators that are derived from polyunsaturated lipids, such as DHA and EPA [6]. Lower extremity artery disease (LEAD) is an atherosclerotic occlusive disease of the lower extremities, where there is a partial or complete obstruction of peripheral arteries, and ROS, despite causing damage to tissues at a high level, if maintained at a low level, plays an interesting role in the treatment of LEAD, such as angiogenesis [7]. Kim et al. have thoroughly explained the causative relationship between oxidative stress and blood–brain barrier (BBB) diseases such as stroke, traumatic brain injury, and Alzheimer’s disease (AD). ROS have played a critical role in modulating the integrity of the tight junction leading to BBB disruption. They also summarized the beneficial effects of natural polyphenols as a therapeutic strategy against BBB-disruption-induced brain diseases [8]. Taken together, these review articles gave an in-depth discussion of how ROS greatly affects the progress of diseases and how natural antioxidant has the potential to alleviate these diseases.

Moreover, the following original articles have further solidified the beneficial effects of natural antioxidants in preventing cardiovascular and cerebrovascular diseases. Yun et al. have demonstrated that sinapic acid, a phenolic compound, has an antioxidant effect against the oxidative-stress-induced hypertrophic cardiomyocytes via the activation of the mitochondria Sirt3/SOD2 signaling [9]. Histochrome, with strong antioxidant activity and iron-chelating effects, has the potential to be an alternative cardioprotection for patients who have undergone coronary reperfusion therapy through inhibition of ferroptosis in cardiomyocyte death [10]. Air pollution, such as fine dust, is considered a risk factor for the premature development of endothelial cells through an increased level of present oxidative stress leading to CVDs. Shiwakoti et al. suggest that *Humulus lupulus* or Hop plant contains an active component in preventing air-pollution-associated CVDs through reducing the oxidative stress mediated by NADPH oxidase and the local renin–angiotensin system, as shown by downregulation of p22 phox and angiotensin type 1 receptor expression, respectively [11]. Rosmarinic acid, which is a natural polyphenol, was found to have beneficial activity in targeting NADPH oxidase/ROS/PKC-δ/integrin αIIbβ3 signaling that is associated with platelet-induced vascular pathology in AD. The BBB acts as a barrier that protects the brain from the infiltration of various pathogens, and disruption of these could lead to neurodegenerative diseases. ROS was found to be one of the mediators for the breakdown of the BBB, triggering inflammation in the brain [12]. Yuzu and its main component hesperidin, a flavonoid, were shown to ameliorate the ischemia/hypoxia-induced BBB dysfunction by maintaining the BBB integrity [13]. Auraptene, a natural compound from citrus fruits, also mediates BBB dysfunction through maintaining the BBB integrity through increasing the expression of junctional proteins and the upregulated levels of mtUPR mRNA and of mRNAs that encode antioxidant enzymes [14]. Taurine, which is widely distributed in the body, exerts neuroprotection in the CNS to some extent, and the formation of taurine chloramine (Tau-Cl) is found to increase the antioxidant enzymes through scavenging the toxic hypochlorous acid [15]. 4,6′-Anhydrooxysporidinone, which is isolated from the fungus *Fusarium latertium* SSF2, inhibited glutamate-induced HT22 cell death related to the accumulation of ROS through the activation of the glutamate-mediated Nrf2/HO-1 pathway [16]. Epigallocatechin was found to demonstrate a neuroprotective effect against cerebral-ischemia-induced brain cortex injury through the regulation of lipid peroxidation, maintaining appropriate concentration of essential elements, and its antioxidant activity [17].

I would like to acknowledge all the authors who have contributed to this Special Issue “Natural Antioxidant in Cardiovascular and Cerebrovascular Diseases”. This Special Issue has provided new insights into the importance of natural antioxidants in the cardiovascular and cerebrovascular diseases, as well as the need to further develop therapeutic strategies targeting these diseases.

## References

[1] Tong X., Yang Q., Ritchey M.D., George M.G., Jackson S.L., Gillespie C., Merritt R.K. (2019). The Burden of Cerebrovascular Disease in the United States. Prev. Chronic Dis..

[2] Kim H.C. (2021). Epidemiology of cardiovascular disease and its risk factors in Korea. Glob. Health Med..

[3] Woo S.H., Kim J.C., Eslenur N., Trinh T.N., Do L.N.H. (2021). Modulations of Cardiac Functions and Pathogenesis by Reactive Oxygen Species and Natural Antioxidants. Antioxidants.

[4] Lee Y., Im E. (2021). Regulation of miRNAs by Natural Antioxidants in Cardiovascular Diseases: Focus on SIRT1 and eNOS. Antioxidants.

[5] Uddin M.J., Kim E.H., Hannan M.A., Ha H. (2021). Pharmacotherapy against Oxidative Stress in Chronic Kidney Disease: Promising Small Molecule Natural Products Targeting Nrf2-HO-1 Signaling. Antioxidants.

[6] Kang G.J., Kim E.J., Lee C.H. (2020). Therapeutic Effects of Specialized Pro-Resolving Lipids Mediators on Cardiac Fibrosis via NRF2 Activation. Antioxidants.

[7] Hutchings G., Kruszyna L., Nawrocki M.J., Strauss E., Bryl R., Spaczynska J., Perek B., Jemielity M., Mozdziak P., Kempisty B. (2021). Molecular Mechanisms Associated with ROS-Dependent Angiogenesis in Lower Extremity Artery Disease. Antioxidants.

[8] Kim Y., Cho A.Y., Kim H.C., Ryu D., Jo S.A., Jung Y.S. (2022). Effects of Natural Polyphenols on Oxidative Stress-Mediated Blood-Brain Barrier Dysfunction. Antioxidants.

[9] Yun U.J., Yang D.K. (2020). Sinapic Acid Inhibits Cardiac Hypertrophy via Activation of Mitochondrial Sirt3/SOD2 Signaling in Neonatal Rat Cardiomyocytes. Antioxidants.

[10] Hwang J.W., Park J.H., Park B.W., Kim H., Kim J.J., Sim W.S., Mishchenko N.P., Fedoreyev S.A., Vasileva E.A., Ban K. (2021). Histochrome Attenuates Myocardial Ischemia-Reperfusion Injury by Inhibiting Ferroptosis-Induced Cardiomyocyte Death. Antioxidants.

[11] Shiwakoti S., Adhikari D., Lee J.P., Kang K.W., Lee I.S., Kim H.J., Oak M.H. (2020). Prevention of Fine Dust-Induced Vascular Senescence by Humulus lupulus Extract and Its Major Bioactive Compounds. Antioxidants.

[12] Lee B.K., Jee H.J., Jung Y.S. (2021). Abeta1-40-Induced Platelet Adhesion Is Ameliorated by Rosmarinic Acid through Inhibition of NADPH Oxidase/PKC-delta/Integrin alphaIIbbeta3 Signaling. Antioxidants.

[13] Lee B.K., Hyun S.W., Jung Y.S. (2020). Yuzu and Hesperidin Ameliorate Blood-Brain Barrier Disruption during Hypoxia via Antioxidant Activity. Antioxidants.

[14] Lee M.J., Jang Y., Zhu J., Namgung E., Go D., Seo C., Ju X., Cui J., Lee Y.L., Kang H. (2021). Auraptene Enhances Junction Assembly in Cerebrovascular Endothelial Cells by Promoting Resilience to Mitochondrial Stress through Activation of Antioxidant Enzymes and mtUPR. Antioxidants.

[15] Seol S.I., Kim H.J., Choi E.B., Kang I.S., Lee H.K., Lee J.K., Kim C. (2021). Taurine Protects against Postischemic Brain Injury via the Antioxidant Activity of Taurine Chloramine. Antioxidants.

[16] Lee D., Choi H.G., Hwang J.H., Shim S.H., Kang K.S. (2020). Neuroprotective Effect of Tricyclic Pyridine Alkaloids from Fusarium lateritium SSF2, against Glutamate-Induced Oxidative Stress and Apoptosis in the HT22 Hippocampal Neuronal Cell Line. Antioxidants.

[17] Lin M.C., Liu C.C., Lin Y.C., Hsu C.W. (2022). Epigallocatechin Gallate Modulates Essential Elements, Zn/Cu Ratio, Hazardous Metal, Lipid Peroxidation, and Antioxidant Activity in the Brain Cortex during Cerebral Ischemia. Antioxidants.

